# Exercise-induced stress behavior, gut-microbiota-brain axis and diet: a systematic review for athletes

**DOI:** 10.1186/s12970-016-0155-6

**Published:** 2016-11-24

**Authors:** Allison Clark, Núria Mach

**Affiliations:** 1 Health Science Department, Open University of Catalonia (UOC), 08035 Barcelona, Spain; 2 Animal Genetics and Integrative Biology unit (GABI), INRA, AgroParis Tech, Université Paris-Saclay, 78352, Jouy-en-Josas, France

**Keywords:** Athlete, Behaviour, Diet, Exercise, Microbiota, Neurotransmitters, Stress

## Abstract

Fatigue, mood disturbances, under performance and gastrointestinal distress are common among athletes during training and competition. The psychosocial and physical demands during intense exercise can initiate a stress response activating the sympathetic-adrenomedullary and hypothalamus-pituitary-adrenal (HPA) axes, resulting in the release of stress and catabolic hormones, inflammatory cytokines and microbial molecules. The gut is home to trillions of microorganisms that have fundamental roles in many aspects of human biology, including metabolism, endocrine, neuronal and immune function. The gut microbiome and its influence on host behavior, intestinal barrier and immune function are believed to be a critical aspect of the brain-gut axis. Recent evidence in murine models shows that there is a high correlation between physical and emotional stress during exercise and changes in gastrointestinal microbiota composition. For instance, induced exercise-stress decreased cecal levels of *Turicibacter* spp and increased *Ruminococcus gnavus,* which have well defined roles in intestinal mucus degradation and immune function.

Diet is known to dramatically modulate the composition of the gut microbiota. Due to the considerable complexity of stress responses in elite athletes (from leaky gut to increased catabolism and depression), defining standard diet regimes is difficult. However, some preliminary experimental data obtained from studies using probiotics and prebiotics studies show some interesting results, indicating that the microbiota acts like an endocrine organ (e.g. secreting serotonin, dopamine or other neurotransmitters) and may control the HPA axis in athletes. What is troubling is that dietary recommendations for elite athletes are primarily based on a low consumption of plant polysaccharides, which is associated with reduced microbiota diversity and functionality (e.g. less synthesis of byproducts such as short chain fatty acids and neurotransmitters). As more elite athletes suffer from psychological and gastrointestinal conditions that can be linked to the gut, targeting the microbiota therapeutically may need to be incorporated in athletes’ diets that take into consideration dietary fiber as well as microbial taxa not currently present in athlete’s gut.

## Background

Stress is an essential adaptation necessary for homeostasis, performance and survival [1]. The stress response occurs whenever an individual is faced with an endogenous or exogenous challenge perceived as unpleasant, adverse or threatening. It can be induced by physical, physiological or psychological stimuli [1]. Intense exercise implies adaptive processes involving affective, physiological, biochemical, and cognitive-behavioral response in an attempt to regain homeostasis (reviewed by Morgan et al [2]). Therefore, it is difficult to differentiate between the effects of the physical stress of exercise and the effects of the psychological stress during exercise [3]. Therefore, both the physical and psychological demands during intense exercise are referred to here as “stress”. According to the review of Purvis et al [4], an estimated 20-60% of athletes suffer from the stress caused by excessive exercise and inadequate recovery. The prevalence of stress is believed to be higher in endurance sports such as swimming, rowing, cycling, triathlon and to some extent long-distance running where athletes are training 4–6 hours a day, 6 days a week, for several weeks without taking time off from intense training [5]. Although there is no consensus as to which symptoms or biomarkers define stress, some common signs that are widely accepted in the scientific literature include clinical, hormonal indicators and other symptoms associated with fatigue, performance decline, insomnia, change in appetite, weight loss and mood disturbances such as irritability, anxiousness, loss of motivation, poor concentration and depression, as well as inflammation and immunosuppression (reviewed by Purvis et al [4]). 

**Fig. 1:**
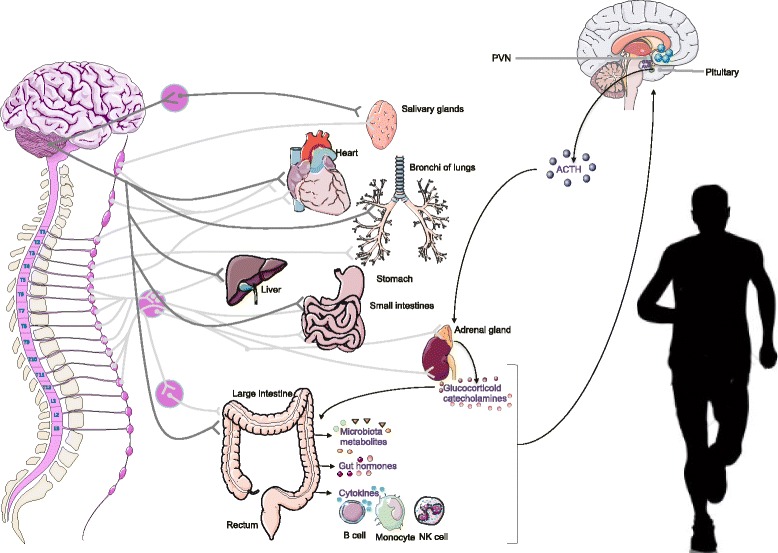
Stress hormones released during high intense exercise. Stress responses to intense exercise are mediated by largely overlapping circuits in the limbic forebrain, the hypothalamus and the brainstem, so that the respective contributions on the neuroendocrine and autonomic systems are tuned in accordance with stressor and intensity [6]. When brainstem receives inputs that signal major homeostatic perturbations, such as respiratory distress, energy imbalance, desydration, visceral or somatic pain, inflammation or exteroceptive factors respond through a coordinated modulation of the HPA axis and the sympathetic and parasympathetic branch of the autonomic nervous system (ANS). By contrast, forebrain limbic regions have no direct connections with the HPA axis or the ANS and thus require intervening synapses before they can access autonomic or neuroendocrine neurons (top-down regulation) [6]. Briefly, exercise-induced stress results in activation of preganglionic sympathetic neurons in the intermediolateral cell column of the thoracolumbar spinal cord (shown in purple and clear grey). This sympathetic activation represents the classic 'fight or flight' response and it generally increases circulating levels of catecholamines. Parasympathetic tone can also be modulated during stress (shown in dark grey color). Parasympathetic actions are generally opposite to those of the sympathetic system and alter the vagal tone to the heart and lungs. Within the HPA axis, stress activates hypophysiotropic neurons in the paraventricular nucleus of the hypothalamus (PVN) that secrete releasing hormones, such as corticotrophin-releasing hormone (CRH) and arginine vasopressin (AVP), into the portal circulation of the median eminence. These releasing hormones act on the anterior pituitary to promote the secretion of adrenocorticotropic hormone (ACTH), which in turn acts on the inner adrenal cortex to initiate the synthesis and release of glucocorticoid hormones. Moreover, the adrenal cortex is directly innervated by the sympathetic nervous system, which can also regulate corticosteroid release. Additionally, gastrointestinal tract responds to stress in an endocrine manner by releasing hormones such as Gamma-amino butyric acid (GABA), neuropeptide Y and dopamine that have been purported to be involved in the gastrointestinal disturbances, anxiety, depression, reduced food intake and less stress coping. Microorganisms that colonize the digestive tract can be involved in the regulation of the HPA axis through the regulation or production of short chain fatty acids and neurotransmitters such as GABA, dopamine and serotonin, as well as cytokines. The neuroendocrine stress response to exercise is determined not only by the emotional stress but the volume of physical exposure, where volume consists of the intensity and/or duration of the exercise session. As exercise intensity is increased, there are approximately proportional increases in circulating concentrations of ACTH and cortisol. There is a critical threshold of exercise intensity that must be reached (~50–60% of maximal oxygen uptake [V_O2max_]) before circulating levels increase in response to exercise [170, 171]

Two main distinct but interrelated systems that affect the stress response during exercise are: the sympatho-adrenomedullary (SAM) and hypothalamus-pituitary-adrenal (HPA) axes. The activation of these axes results in the release of catecholamines (norepinephrine (NE) and epinephrine) and glucocorticoids into circulatory system (reviewed by Ulrich-Lai et al [6] (Fig. 1). Stress during exercise also activates the autonomic nervous system (ANS) [7], which provides the most immediate response to stressor stimulus through its sympathetic and parasympathetic arms, and increases the neuronal release of NE and other neurotransmitters in peripheral tissues such as the gastrointestinal (GI) tract or cardiovascular system (extensively reviewed by Ulrich-Lai et al [6]). The bidirectional communication between the ANS and the enteric nervous system (ENS) in the GI tract, the gut-brain axis, mainly occurs by way of the vagus nerve, which runs from the brain stem through the digestive tract and regulates almost every aspect of the passage of digested material through the intestines (reviewed by Eisenstein [8]). Other ways of communications between the gut-brain axis are: (i) gut hormones [9] (i.e. gamma aminobutyric acid (GABA), neuropeptide Y, dopamine) and (ii) gut microbiota molecules [10, 11] (i.e. short chain fatty acids (SCFA), tryptophan).

The human gut harbors more than 100 trillion microorganisms in the GI tract, which represents roughly 9 million genes [12]. Overall, the gut microbiota comprises five phyla and approximately 160 species in the large intestine [13]. The gut microbiota promotes digestion and food absorption for host energy production [14–16] and provide folate [17], vitamin K_2_ [18] and SCFAs [19]. In the human large intestine, complex carbohydrates are digested and subsequently fermented by the anaerobic intestinal microbiota into SCFAs such as N-butyrate, acetate, and propionate (eloquently reviewed by Flint et al [19]). The gut microbiota also neutralizes drugs and carcinogens, modulates intestinal motility, protects the host from pathogens, stimulates and matures the immune system and epithelial cells (reviewed by Nicholson et al [20]). Evidence shows that the gut microbiota also modulates excitatory and inhibitory neurotransmitters (i.e. serotonin, GABA and dopamine) and neurotransmitter-like substances, especially in response to physical and emotional stress (reviewed by Clarke et al. [21] and Moloney et al [22]). A systematic review on endurance exercise and gut microbiota [23] suggested that gut microbiota might have a key role in controlling the oxidative stress and inflammatory responses as well as improving metabolism and energy expenditure during intense exercise. However, beyond those functions, we noted that the relationship between exercise-induced stress and gut microbiota composition, as well as the possible pathophysiological mechanisms involved has not yet been explored.

New research has shown that diet can greatly influence the gut microbiota composition, which can greatly impact host's health (see review by Fasano [24]). Dietary changes can account for up to 57% of gut microbiota changes, whereas genes account for no more than 12% [25]. Short term consumption of a mostly animal or mostly plant diet can dramatically alter the microbiota composition and function [26], as fast as 24 hours [27]. The general guidelines of American Dietetic Association (ADA) [28] for meals and snacks in athletes recommend high amounts of simple carbohydrates intake (6 to 10 g/kg per day) to maintain blood glucose and maximize glycogen stores, high to moderate amounts of animal protein intake (1.2 to 1.7 g/kg per day) to satisfy the muscle accretion needs, low amounts of fat intake (20-35% of the dietary energy) and low amounts of fiber intake to facilitate gastric emptying and minimize gastrointestinal distress [28]. The insufficient consumption of fiber and resistant starch may promote a ‘loss’ of microbiota diversity and function in the GI [26].

Given the interaction between the gut microbiota and gut-brain axis upon stress, and its interaction with food consumed, the aim of this systematic review is to summarize the available evidence supporting the interactions between exercise-induced stress responses and the gut microbiota, as well as its possible effects on the health and performance of the elite athletes. A secondary aim is to define dietary strategies that could modify the microbiota composition and improve both overall health, (i.e. improving the conditions of the intestinal epithelium, the immune system response or the stress response), and performance (i.e. improving energy availability from diet and controlling the inflammation levels in athletes).

## Main text

### Role of microbiota in controlling hormone release associated with exercise-induced stress

Elite athletes who train and compete for hours experience physical and emotional stress that causes shifts in physiological homeostasis stimulating the SAM and HPA axis [2] (Fig. 1). As reviewed by Ulrich-Lai et al [6], the SAM system, which is part of the sympathetic division of the autonomic nervous system, releases epinephrine from the medulla or center of the adrenal gland, which facilitates rapid mobilization of metabolic resources and regulation of the fight/flight response. It generally increases circulating levels of adrenaline (primarily from the adrenal medulla) and noradrenaline (primarily from sympathetic nerves), heart rate and force of contraction, peripheral vasoconstriction, and energy mobilization [6]. Parasympathetic tone can also be modulated during stress [6] (Fig. 1).

On the other hand, stress stimuli activates the hypothalamic paraventricular nucleus (PVN) that interconnects with the bed nucleus of the stria terminalis (BNST) [6]. These neurons synthesize corticotropin-releasing hormone (CRH) and arginine vasopressin (AVP), which are released into the hypophysial portal circulation and transported to the anterior pituitary gland, where they stimulate the release of adrenocorticotropin (ACTH) into the systemic circulation [6]. ACTH interacts with receptors on the cortex of the adrenal gland to stimulate the production and release of glucocorticoids (GCs) into general circulation [6]. The effect of GCs depends upon the receptors to which they bind. There are two GC receptors: mineralocorticoid receptor (MR) and glucocorticoid receptor (GR). Outside the brain, GCs operate through GRs, whereas in the brain, GCs bind to both MR and GR (reviewed by Ulrich-Lai et al [6]). GRs mediate most of the stress effects of glucocorticoids (including metabolism and immunity). By binding to the GR, GCs inhibit the further release of CRH, thereby regulating both the basal HPA tone and the termination of the stress response [6]. MRs foster cellular activation (hippocampus) and mediate most of the basal effects, which include maintaining responsiveness of neurons to their neurotransmitters, maintaining the HPA circadian rhythm and maintaining blood pressure [29].

Acute physical exertion above 60% maximal oxygen uptake (V_O2max_) is one of the physical stresses that stimulates the HPA axis and release of stress and catabolic hormones [30], whereas exercising below this intensity fails to cause such a spike in serum cortisol [31]. Exercising at 80% capacity has been shown to provoke a significant increase in ACTH levels pre- and post-exercise [31]. Furthermore, comprehensive studies in endurance athletes conducted by Lehmann [32, 33] during the past 20 years have shown that 60-80% of athletes in the early stage of chronic stress have higher pituitary CRH-stimulated ACTH response. Therefore, there is a clear connection between exercise-induced stress and increased stress hormone levels in athletes.

Stress during exercise also activates the ANS [7], which increases the neuronal release of NE and other neurotransmitters in peripheral tissues such as the GI tract. Exercise and gut symptomatology have long been connected (reviewed by Cronin et al [34]). The bidirectional communication between the ANS and the ENS in the GI tract, the gut-brain axis, mainly occurs by way of the vagus nerve, which runs from the brainstem through the digestive tract (reviewed by Carabotti [35]). Beyond neuronal connection, other ways of communications between the gut-brain axes are via gut hormones [9] and gut microbiota molecules [10, 36]).

There is increasing evidence that GI tract responds to stress by releasing hormones such as GABA, neuropeptide Y (NPY) and dopamine (reviewed by Holzer [37]). GABA, which is the body's dominant inhibitory neurotransmitter of the CNS, regulates blood pressure and heart rate and plays a major role in various gastrointestinal functions such as motility, gastric emptying and transient lower esophageal sphincter relaxation, as well as anxiety, depression, pain sensation and immune response [38]. Moderate exercise can increase GABA levels in the hypothalamus resulting in lower resting blood pressure, heart rate and sympathetic tone [39]. Under forced swimming in 25 °C water, de Groote and Linthorst [40] found that hippocampal GABA levels in rats decreased (70% of baseline). However, to discriminate between the psychological and physical aspects (i.e. the effects on body temperature) of forced swimming, another group of animals was forced to swim at 35 °C [40]. This later stressor, like novelty, caused an increase in hippocampal GABA (120% of baseline), suggesting a stimulatory effect of psychological stress [40].

NPY is also released in response to various stress stimuli, such as intense exercise, in the GI tract and plays a role in attenuates the HPA axis [41]. The NPY is a 36-amino acid peptide located throughout the gut-brain axis and is the most prevalent neuropeptide in the brain that plays a role in stress resilience, and inflammatory processes [42]. Rämson et al [41] studied NPY serum levels in 12 highly trained rowers and found that the post-exercise concentrations of NPY increased significantly. Though few studies have studied serum and hippocampal NPY levels in response to exercise, these results suggest it plays a role in reducing the stress response upon intense exercise [41].

Lastly, dopamine, the precursor to NE and epinephrine, can also be synthesized during stress in the GI tract. Dopamine production is dependent upon several factors: levels of its precursor tyrosine, enteric bacteria that directly produce dopamine, the type of stress experienced and sex [43]. There are several dopamine receptors throughout the intestines suggesting it plays a role in the gut-brain axis [43]. The GI tract, spleen and pancreas produce substantial amounts of dopamine [43]. The rate-limiting enzyme for dopamine synthesis, tyrosine hydroxylase, is found in human stomach epithelial cells showing its function exist beyond neurotransmission in the brain [43]. Habitual physical activity for about 1–2 hours a day has been shown to increase dopamine levels in the brain [44].

Recent studies and literature about gut-brain axes have focused on the role of microbiota and its molecules on controlling anxiety and depression (reviewed by Foster [45]). However, the role of microbiota in controlling exercise-induced stress adaptation remains unknown. The use of germ-free (GF) animals has provided one of the most significant insights into the role of the microbiota in regulating the development and function of the HPA axis in response to stress [21]. In GF mice, a mild restraint stress induced an exaggerated release of corticosterone and ACTH compared to the specific pathogen free (SPF) controls, thus elucidating a connection between the gut microbiota and HPA axis [46]. This aberrant stress response in GF mice was partially reversed by colonization with fecal matter from SPF animals and fully reversed by mono association of *Bacillus infantis* in a time dependent manner [47]. Thus, the gut microbial composition is critical to the development and function of an appropriate stress response and HPA axis [47]. In addition, there is increasing evidence shows that the commensal and resident community of gut microorganisms can regulate the HPA axis through the synthesis of hormones and neurotransmitters such as GABA, dopamine and serotonin (Table 1). Asano et al [48] discovered that SPF mice had substantially higher levels of free, biologically active dopamine and NE in the gut lumen of the ileum and colon than GF mice. Moreover, GF mice treated with *Clostridium* species, fecal flora from the SPF mice or *E. coli* showed elevated levels of free catecholamines suggesting that the gut microbiota plays a role in their synthesis through dopamine regulation [48]. Moreover, other mouse studies suggest that the vagus nerve serves as some sort of 'hotline' by which gut microbes communicate directly with the CNS [8]. For instance, Bravo et al [49] have found that *Lactobacillus* strain affects the CNS by regulating emotional behavior and central GABA receptor expression via the vagus nerve. Given the apparent link between early-life events and subsequent adult neurogenesis response to stress [50], researchers need to understand whether the potential effects of disruptions to the microbiota in childhood might affect the neurobiology of stress and endocrine function of the microbiota. What is still missing is solid evidence that demonstrates gut microbiota causality in stress [8].

**Table 1:** Bacterial strains that affect neurotransmitter and stress hormone production- an update from Clarke et al [21]

Molecule	Probiotic Strain, microbial metabolite	Species	Effects
Tryptophan- precursor to 5-HT	*Bifidobacterium infantis*	Rats	Aids in combating psychiatric disorders such as depression [84]
	*Lactobacillus johnsonii*	In vitro model	Reduces serum kynurenine concentrations and IDO activity in vitro in HT-29 colonic cells, which prevents the breakdown of tryptophan [138]
Serotonin	*Lactococcus lactis subsp. Cremoris (MG 1363), Lactococcus lactis subsp. Lactis (IL 1403), Lactobacillus plantarum (FI8595), Streptococcus thermophilus* (NCFB2392), *Eschericchia coli* K-12, *Morganella morganii* (NCIMB, 10466), *Klebisella pneumoniae (*NCIMB, 673), *Hafnia alvei* (NCIMB, 11999)	In vitro model	Produce serotonin [47, 139]
	Butyrate and acetate produced by bacteria	Mice	Induce serotonin synthesis in a dose-dependent manner by regulating the gene Tph1 that synthesizes serotonin [68]
Dopamine	*Bacillus cereaus, Bacillus mycoides, Bacillus subtilis, Proteus vulgaris, Serratia marcescens, S. aureus, E.col, E.coli* K-12, *Morganella morganii* (NCIMB, 10466), *Klebisella pneumoniae (*NCIMB, 673), *Hafnia alvei* (NCIMB, 11999)	In vitro model	Produce dopamine [140–142]
GABA	*Lactobacillus rhamnosus (JB-1)*	Mice	Regulates GABA receptor expression and reduced stress-induced corticosterone, anxiety and depression [143]
	*Bifidobacterium dentium* DPC6333*, Bifidobacterium dentium* NCFB2243*, B. infantis* UCC35624, *Bifidobacterium adolescentis* DPC6044*, Lactobacillus brevis* DPC6108*, Bacillus mycoides, Bacillus subtiles, Proteus vulgaris, Lactobacillus rhamnosus* YS9	Humans	Lactobacillus brevis DPC6108 was the most effective at producing GABA [8].
Cortisol	*Lactobacillus helveticus* R0052*; Bacteroidetes longum* R0175	Humans	Reduce urinary free cortisol output [133, 144]
Noradrenaline	*B. mycoides, B. subtilis, P. vulgaris, S. marescens, E. coli* K-12	In vitro chromatography	Regulates motility and secretions in the ENS. Elevated levels due to acute stress can cause the growth of pathogenic *E. coli* [140].

Of growing interest is how the gut microbiota interact directly with stress hormones in peripheral tissues such as the mucosal layer of the GI tract, which is called microbial endocrinology [10]. NE has shown to have a direct effect on gut *Aeromonas hydrophila, Bordetella spp., Campylobacter jejuni, Helicobacter pylori, Listeria spp.* and *Salmonella enterica spp.* among others. Some of the ways that NE can promote pathogenic bacterial growth is by facilitating *E. coli* to adhere to the intestinal wall by increasing the expression of its virulence factor K99 pilus adhesin as well as activating the expression of virulence-associated factors in *Salmonella typhimurium,* which then makes infection by these bacteria easier [10]. Additionally, NE has also been shown to increase levels of non-pathogenic *E.coli* and other gram-negative bacteria [51].

Currently, only one study has shown that exercise-induced stress directly modifies gut microbiota composition in non-GF or SPF animals. Allen et al [52] recently published the first study that increases the understanding of how the microbiome regulates the exercise-induced stress response, revealing unique microbiota-host interactions that are important for gastrointestinal and systemic health [52]. Voluntary wheel running for 6 weeks attenuated symptoms, whereas forced treadmill running exacerbates intestinal inflammation and clinical outcomes in a colitis mouse model [52]. Fecal and cecal levels of *Turicibacter* spp., which has been strongly associated with immune function and bowel disease, were significantly lower in voluntary runners compared to the 6 week forced treadmill running group. Additionally, *Ruminococcus gnavus,* which has well defined roles in intestinal mucus degradation, was increased in the forced group compared to sedentary group [52], together with *Butyrivibrio* spp., *Oscillospira* spp., and *Coprococcus* spp. This preliminary study in exercised and stressed-animals shows that physical activity can alter microbiota composition as well as metabolic function that could either positively or negatively affect performance.

### Role of microbiota in controlling gastrointestinal symptoms associated with exercise-induced stress

**Fig. 2:**
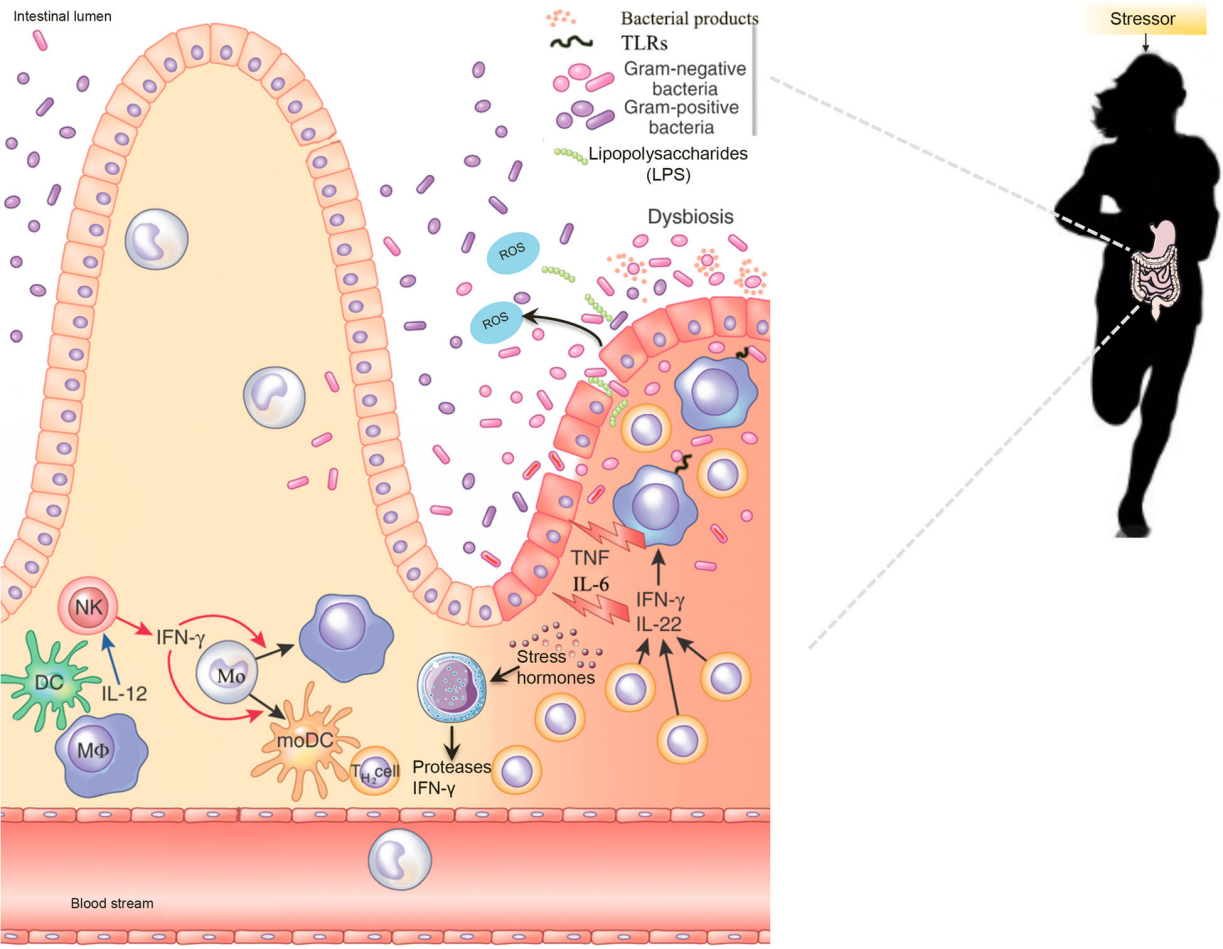
Gastrointestinal disruption during high intensity exercise. Proper intestinal barrier function is crucial for maintaining health and immunity. During intense exercise, athletes’ body temperature increases and blood pools away from the gastrointestinal tract to periphery muscles and organs such as the heart and lungs during intense physical activity [62]. The redistribution of blood flow away from the intestines together with thermal damage to the intestinal mucosa can cause intestinal barrier disruption, followed by an inflammatory response [63]. Additionally, intense exercise over a prolonged period of time increase stress hormones and lipopolysaccharides (LPS) translocation in the gastrointestinal tract, which triggers immune responses that often results in increased pro-inflammatory cytokines and intestinal permeability. Additionally, intestinal permeability may be made worse by the increased production of reactive oxygen species (ROS) and by the alteration of gut-microbiota composition and activity (the so-called dysbiosis). Furthermore, gastrointestinal tract responds to stress by releasing hormones such as GABA, neuropeptide Y (NPY) and dopamine that have been purported to cause GI disturbances, anxiety, depression, reduced food intake and less stress coping [9]. Conversely, the microbiota’s production of butyrate and propionate can increase transepithelial resistance, which improves intestinal barrier function and decreases inflammation.

Proper intestinal barrier function is crucial for maintaining immune and overall health [53]. There are more than 50 proteins that play an important role in regulating the tight junctions of the mucosal endothelial layer and thus intestinal permeability [54]. The tight junction complexes consist of 4 trans membrane proteins: occludin, claudins, junctional adhesion molecules and tricellulin that interact with the structural zonula occludens proteins (ZO1, ZO2 and ZO3) [54]. Under normal conditions, the tight junction complexes work to maintain the polarization of the intestinal barrier that controls the paracellular passage of only small molecules such as ions, water and leukocytes [54]. The intestinal barrier also serves as a doorway between the microorganisms and their byproducts, the enteric immune system response and the nutrient particles inside and outside the GI tract (Fig. 2) [55]. As detailed in a review based on acute effects of exercise on immune and inflammatory indices in untrained adults [56], increased intestinal permeability, or “leaky gut” as it is commonly called, is a loosening of the tight junction protein structures. An excessive release of stress hormones induced by physical and psychological stress can cause lipopolysaccharides (LPS) translocation outside of the GI tract triggering immune and inflammatory responses often resulting in increased intestinal permeability [56]. The translocated LPS are detected by CD14 and toll-like receptor 4 (TLR4), which causes the release of pro-inflammatory cytokines such as tumor necrosis factor alpha (TNFα), interferon alpha (IFNα), interferon-gamma (INFγ) and interleukins (IL1β or IL6), which can eventually result in endotoxemia [57] (Fig. 2). These pro-inflammatory cytokines also increase the opening of the tight junctions through ZO1 and ZO2 pathways of tight junction protein complexes that can result in endotoxemia [57]. Additionally, the activation of the HPA axis may stimulate subepithelial mast cells to secrete immune mediators such as histamine, proteases and pro-inflammatory cytokines [58], trigging intestinal permeability [59].

Depending on the type of exercise, intensity, age and other factors, between 20-50% of athletes suffer gastrointestinal symptoms, which have been shown to increase with exercise intensity [60]. In a study of 29 highly trained male triathletes, Jeukendrup et al [61] discovered that upon competition, 93% reported digestive disturbances and two participants had to abandon the race because of severe vomiting and diarrhea. According to expert review [53], hyperthermia, ischemia and hypoperfusion are other severe stimuli that can cause a loosening of the tight junctions during intense exercise. These are common occurrences among athletes as body temperature increases and blood pools away from the GI tract to periphery muscles and organs such as the heart and lungs during intense physical activity [62]. The redistribution of blood flow away from the intestines together with thermal damage to the intestinal mucosa can cause intestinal barrier disruption, followed by an inflammatory response [63]. In healthy young adult male cyclists who performed endurance sports activities during 4–10 hours per week, just one hour of physical activity at 70% maximum workload capacity produced splanchnic hypoperfusion, which can cause decreased GI circulation, increased intestinal permeability and damage to the small intestine [64]. Another study showed humans exercising at 70% V_O2max_ presented a 60-70% reduction in splanchnic blood flow, and exercise-induced ischemia caused increased intestinal permeability when blood flow was reduced by 50% [65]. Ischemia also increases reactive oxygen species (ROS) production, which induces leaky gut as activated protein kinases phosphorylate tight junctions proteins resulting in hyperpermeability (Fig. 2) [57]. Hydrogen peroxide can also serve as a signaling molecule that activates transcription of several pro-inflammatory genes including nuclear factor kappa-light-chain-enhancer of activated B cells (NFKβ), TNFα, IL6, IFNγ, and IL1β [65] that can compromise barrier function. Therefore hypoperfusion and ischemia can result in increased intestinal permeability opening the door for LPS and enteric bacteria to circulate the bloodstream, possibly leading to endotoxemia. For instance, marathon, triathlete and ultra endurance athletes have been reported to have plasma LPS concentrations of 5 to 284 pg/mL along with up to 93% of athletes reporting digestive disturbances, which could be caused by the LPS-induced cytokine response [61]. Brock-Utne et al [66] discovered that 81% of randomly selected exhausted marathon runners showed endotoxemia (0,1 ng/mL), 2% presented lethal levels above 1 ng/mL and only 19% had normal levels. In addition, 58 out of the 72 runners who experienced high LPS levels also suffered from GI upset such as nausea, diarrhea and/or vomiting, whereas only 3 out of the 17 runners who had low plasma endotoxin concentrations reported such symptoms. The marathon participants who took more than 8 hours to complete the race suffered higher plasma endotoxin concentrations [66]. In a study in 18 triathletes who competed in an ultra triathlon that was 90 km long, their mean plasma LPS concentrations increased from 0.081 to 0.294 ng/mL, and their mean plasma anti-LPS immunoglobulin G concentrations decreased from 67.63 to 38.99 μg/mL. Starkie et al. [67] studied heat stress and immune response after intense cycling in seven healthy male athletes. Glucocorticoids released during intense exercise has also been shown to diminish the TLR expression, and therefore the capacity to produce anti-inflammatory cytokines and host antimicrobial defense [68]. All these studies show that intense exercise during prolonged periods not only can lead to increased intestinal permeability and thus increased plasma LPS levels, but it can also cause immunosuppression [69].

Given the gut microbiota’s diverse role in GI function, enteric immunity [70], endocrinology [11] as well as regulating oxidative stress [71–73] and hydration levels, it is not surprising that efforts to identify the mechanisms by which gut microbiota improves intestinal barrier function of elite athletes are increasing.

In the colon and cecum, complex plant-derived polysaccharides are digested and subsequently fermented by gut microorganisms, such as *Lactobacillus, Bifidobacterium, Clostridium, Bacteroides*, into SCFAs and gases which are also used as carbon and energy sources by specialized bacteria such as reductive acetogens, sulfate-reducing bacteria and methanogens (reviewed by Marchesi et al [36] and by Flint et al [19]). Acetate, propionate, and N-butyrate are present at a molar ratio of approximately 60:20:20 in the colon and feces [74]. The composition of the gut microbiota, metabolic interactions between microbial species [52] and the amount and type of the main dietary macro- and micronutrients determine the types and amount of SCFAs produced by gut microorganisms [75, 76]. The more plant-derived polysaccharides, oligosaccharides, resistant starch and dietary fiber one eats, the more these bacteria can ferment these indigestible food sources into beneficial SCFA. The microbiota-produced SCFAs affect a range of host processes including control of colonic pH, with consequent effects on microbiota composition, intestinal motility, gut permeability and epithelial cell proliferation [77]*.* N-butyrate produced by gut bacteria regulates neutrophil function and migration, inhibits inflammatory cytokine-induced expression of vascular cell adhesion molecule-1, increases expression of tight junction proteins in colon epithelia and exhibits anti-inflammatory effects (reviewed by Nicholson [20])_._ N-butyrate and propionate can increase transepithelial resistance which improves intestinal barrier function and decreases inflammation [78]. They also serve as a primary energy source, about 60-70%, for colonocytes [74], which prevents mucosal degradation [79] that can occur as a result of intense exercise due to hypoperfusion and ischemia for example. Matsumoto et al [80] performed a study in 14 male Wistar rats during five weeks. The control group was sedentary and the exercise group had access to an exercise wheel in their cage. They discovered through 16S rRNA gene sequencing that the rats that voluntarily exercised using the wheel presented higher cecum levels of SCFA than the sedentary control group. Levels of N-butyrate increased significantly between the exercise groups (8.14 ± 1.36 mmol/g of cecal contents) compared to the control group (4.87 ± 0.41 mmol/g of cecal contents) [80]. The cecum was approximately 1.5 times larger in the exercise group than in the control group, and the cecal tissue weights and contents were much greater in the exercise group than in the control group, indicating that a significant change had occurred in the cecal environment in response to voluntary wheel running [80]. Additionally, the cecal microbiota and SCFA profiles were much different between the exercise and control groups [80].

In general, exercise-induced stress can diminish intestinal barrier function and cause LPS translocation which results in GI upset, hydration imbalances, poor uptake of nutrients and electrolytes, as well as thermal damage of the intestinal mucosa, all of which negatively affect athletic performance [64]. Although there are few studies that show the effect exercise has on SCFA levels in the cecum and energy metabolism, those that do exist, illustrate how intense exercise affect SCFA production, which in turn affects the HPA axis, the GI health and may promote favorable athletic performance.

### Role of microbiota in controlling the mood disturbances, fatigue, insomnia and depression associated with exercise-induced stress

Many athletes who suffer from stress enter into a vicious cycle of over exerting themselves with strenuous training and competitions, which results in fatigue causing them to over train in order to overcome fatigue and decreased athletic performance [4]. Some scientists believe that evaluating the athlete's mood is the best way to tell if someone is suffering from stress as it is one of the most common symptoms [81]. To date, several biological mechanisms have been proposed to explain exercise induced mood disturbances, fatigue, insomnia and depression in athletes: (i) metabolic changes in muscle that ultimately lead to muscle exhaustion, and (ii) modifications in the CNS, which are termed central fatigue.

**Fig. 3:**
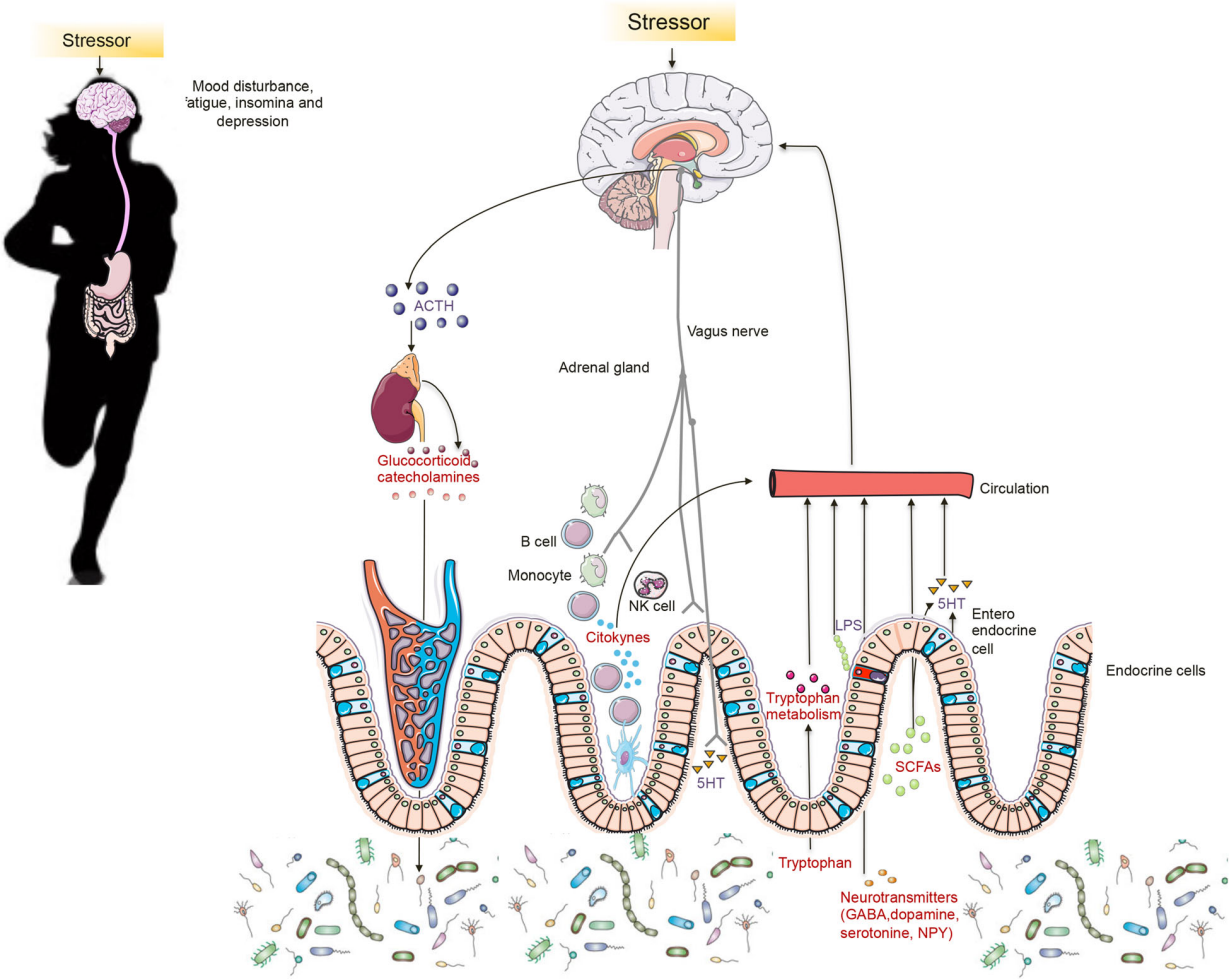
Gut microbiota effects on mood disturbance, fatigue, insomnia and risk of depression during exercise. The putative mechanisms by which bacteria connects with the brain and influence behavior during exercise include bacterial subproducts that gain access to the brain via the bloodstream and the area postrema, via cytokine release from mucosal immune cells, via the release of gut hormones such as 5-hydroxytryptamine (5-HT) from enteroendocrine cells, or via afferent neural pathways, including the vagus nerve. Stress during intense period of training and competitions can influence the microbial composition of the gut through the release of stress hormones or sympathetic neurotransmitters that influence gut physiology and alter the habitat of the microbiota (reviewed by Mach [23]). Alternatively, host stress hormones such as noradrenaline might influence bacterial gene expression or signaling between bacteria, and this might change the microbial composition and activity of the microbiota.

The central fatigue hypothesis states that increased of the neurotransmitter serotonin (5-hydroxytryptamine; 5-HTP) release is associated with sleep, drowsiness and central fatigue, which contribute to suboptimal physical performance (reviewed by Best et al [82], Fig. 3). Furthermore, low serotonin in the brain also causes mood disorders and depression as well as changes in gut transit, blood pressure, cardiac function and platelet aggregation (reviewed by Evans et al [83], Fig. 3). Approximately 95% of the body's serotonin is produced in the enterochromaffin cells (EC) of the intestines [8, 83], which plays a role in enteric motor and sensory functions such as visceral pain perception, further illustrating the gut-brain connection [84]. According to a review conducted by Best et al [82], about 2% of ingested tryptophan is used for the synthesis of serotonin. However, during exercise, serotonin levels might also be increased through other known pathways: (i) kynurenine pathway [21], and (ii) gut microbiota synthesis [85, 86].

Once in the CNS, L-tryptophan is converted from tryptophan hydroxylase (TPH) into 5-HTP, the rate limiting step in brain serotonin synthesis [87]. The 5-HTP is then rapidly decarboxylated by the aromatic amino acid decarboxylase (AADC) to produce cytosolic serotonin [82]. For many years, a single gene encoding 5-TPH was believed to be responsible for serotonin biosynthesis in vertebrates. However, Walther and et al [88] have reported the existence of two distinct TPH genes in humans: TPH1 and TPH2. Of these enzymes, TPH1 is expressed in the periphery and in the pineal body, whereas TPH2 appears to be responsible for the synthesis of serotonin in the rest of the brain. Therefore, TPH could reflect an adaptation to different needs for regulation of serotonin production in the brain and peripheral organs [87].

Serotonin production may also occurs via the kynurenine pathway that is regulated by the tryptophan-degrading enzyme, indoleamine 2,3-dioxygenase (IDO) and tryptophan-2,3-dioxygenase (TDO) [21]. IDO is stimulated by oxidative stress and pro-inflammatory cytokines such as IL6 and TFNα, which are released due to LPS-induced intestinal permeability experienced during intense exercise [89]. On the other hand, glucocorticoids can activate TDO [90, 91], and there is a growing body of evidence suggesting that a hyperactive HPA axis often co-occurs with depression due to increased levels of glucocorticoid hormones, systemic inflammation and increased production of pro-inflammatory cytokines [90] all of which are released due to exercise-induced stress and increased intestinal permeability. Consequently, glucocorticoids and pro-inflammatory cytokines induce TDO and IDO enzymes leading to less serotonin synthesis and possibly fatigue and depression that many athletes who suffer from stress.

A very recent review on the microbiome [8] describes that microbiota also influence production of serotonin (Fig. 3). For instance, a study led by Yano et al. [92] has demonstrated in mice that indigenous spore-forming microbes directly stimulated intestinal serotonin synthesis and release.

When focusing to the interaction between serotonin and exercise, running at low speed appears to increase cerebral serotonin levels and decrease depressive and anxious behavior, whereas high-speed running causes an increase in the gene expression of CRH [93]. Additionally, acute aerobic exercise has been shown to increase 5-HTP levels in the brain stem and hypothalamus in rats after swimming for 30 min/day for 6 days per week for 4 weeks [94]. The rise in brain tryptophan is claimed to result from exercise-induced elevations in serum of non-esterified fatty acid concentrations, which dissociate tryptophan from albumin in the blood and increase the serum free tryptophan (reviewed by Fernstrom and Fernstrom [95]). On the other hand, ample evidence shows that the serum free tryptophan does not dictate brain tryptophan uptake, nor do serum tyrosine levels and branched chain amino acids (BCAA) (i.e. leucine, isoleucine and valine), which is postulated to compete with tryptophan to cross the blood–brain barrier. However, Pechlivanis et al [96] reported different results when analyzing 22 serum metabolites of 14 young athletic men who responded to an intermittent sprint training program involving a very short recovery interval and another program with a longer recovery interval for eight weeks at 80% of V_O2max_. They discovered the leucine, valine and isoleucine decreased after pre-training exercise in both groups, suggesting that BCAA were probably taken up by the muscles during exercise possibly allowing more free tryptophan to cross the blood–brain barrier enabling serotonin synthesis. Of note is that increased lactate levels can also cause fatigue in athletes, not just serotonin synthesis caused by an influx of free tryptophan entering the brain. Nevertheless, Fernstrom et al [95] state that the central fatigue hypothesis is weak because there lacks evidence that shows what specifically causes an increase in brain tryptophan during exercise [95].

In regard to the central fatigue hypothesis, there is overwhelming evidence that the involvement of other molecules could contribute to central fatigue (reviewed by Foley and Fleshner [97] and by Foley [97]). It is suggested that altered dopaminergic pathways involving movement lead to fatigue (reviewed by Foley [97]). Fatigue can set in during exercise when dopamine levels start to drop while serotonin levels are still elevated [98]. The precise mechanisms for how a reduction in brain dopamine could impair exercise performance and influence central fatigue are yet not fully understood. Attempts have been made to prolong dopamine neurotransmission during exercise to fatigue. For example, manipulations of tyrosine and dihydroxyphenylalanine availability are just one instrument used to increase dopamine synthesis during exercise [97]. Further examples are depicted below (section “dietary recommendations to reduce exercise-induce stress behaviour”). Like 5-HTP, dopamine cannot easily cross the blood–brain barrier [97]; therefore, neurons must synthesize dopamine from its precursor tyrosine that’s ingested from the diet [97]. Tyrosine must compete with other amino acids for entry into the brain, including tryptophan and the BCAA, as they are mediated by the same carrier system (reviewed by Foley [97]). However, unlike tryptophan hydroxylase, the brain levels of tyrosine hydroxylase are saturated with substrate under normal conditions, and therefore, any attempt to increase the tyrosine concentrations cannot produce significant increases in dopamine [99].

Moreover, dopamine is an important neurotransmitter associated with motivation and reward [97]. Voluntary wheel running have been rewarding for rats in various experiments, but repeated exposure to natural rewards, like habitual exercise, can modify dopaminergic neuro circuitry negatively altering the motivation and reward centers in the brain associated with exercise resulting in fatigue [97]. On the other hand, moderate aerobic exercise has been shown to increase dopamine levels while reducing serotonin levels in the nigrostriatal tract [44], illustrating that exercise can greatly alter neurotransmitter metabolism.

Other neuromodulators that may influence fatigue and mood during exercise include pro-inflammatory cytokines and ammonia. Increases in pro-inflammatory cytokines like IFNγ and IL6 have been associated with reduced exercise tolerance, acute viral or bacterial infection and increased tryptophan catabolism which could thus limit brain serotonin synthesis [100], leading to depressive behavior. Accumulation of ammonia in the blood and brain during exercise could also negatively affect the CNS function causing fatigue. Guezennec et al [101] investigated if exhaustive exercise increased ammonia detoxification in the brain mediated by glutamine synthesis which, subsequently would influence glutamate and GABA levels. They discovered that both trained and untrained rats that ran until exhausted presented an increase in serum ammonia, which can reduce brain energy by stimulating the Krebs cycle and glycolysis. The trained exercise group had levels of ammonia 50% higher than the untrained group and also presented lower levels of the excitatory neurotransmitter glutamate as well as a decrease in GABA in the striatum of the brain [101]. These findings show that exercise stimulates glutamine synthesis that’s used for ammonia detoxification resulting in decreased production of the excitatory neurotransmitter glutamate possibly causing fatigue in endurance athletes [101]. Glutamine, a nonessential amino acid that’s the most abundant in the human body [102], is crucial not only for the glutamate and GABA synthesis but also for optimal functioning of leukocytes such as lymphocytes and macrophages, T cell proliferation and function [104], intestinal enterocytes growth [102]. Therefore, prolonged intense exercise could negatively affect neurotransmitter homeostasis and immune response when glutamine levels are depleted [80, 81] in order to detoxify ammonia in the brain causing a more excitatory-glutamate driven response to exercise-induced stress and a decline in GABA-mediated inhibitory pathways. Additionally, depleted serum glutamine levels mean less uptake in the intestines leaving the enterocytes more susceptible to intestinal permeability [105].

The influence of gut microbiota on behavior is becoming increasingly evident [85]. As explained above, the gut microbiota serves as an endocrine organ in many ways facilitating the production and regulation of various neurotransmitters and hormones (Table 1), which can affect an athlete’s mood, motivation, and sensation of fatigue (Fig. 3). There is strong evidence that low levels or an absence of gut microbiota (i.e. GF animals) increase tryptophan and serotonin levels and modify central higher order behavior [106]. Desbonnet et al [107] administered the probiotic strain *Bifidobacterium infantis* during 14 days in naive rats who performed a forced swim test. Although the probiotic had no effect on swim performance, there was a significant reduction of IFNγ, TNFα and IL-6 in the probiotic-treated rats compared to controls, and there was a significant increase in plasma concentrations of tryptophan but also kynurenic acid in the bifidobacteria-supplemented rats. GF rats on the other hand had lower levels of tryptophan that increase after administering bifidobacteria species [107]. The authors concluded that this probiotic may have antidepressant effects and illustrates how gut bacteria can ultimately modulate serotonin levels [107]. Moreover, butyrate at the levels of 8 and 16 mM can indirectly affect serotonin synthesis in a dose-dependent manner by regulating the gene TPH1 in EC [108], which reinforce the role that bacteria has on behaviour regulation. More research is needed to show how certain bacteria strains can modulate neurotransmitters metabolism after strenuous activity in humans, while increasing the serum glutamine and the GABA production.

### Dietary recommendations to reduce the exercise-induced stress behavior and symptoms and to improve the gut microbiota composition and function for athletes

Proper training programs aim to balance the systemic stressors that elite athletes experience together with personalized diet plans in order to improve performance and reduce the exercise-induced stress symptoms. Under stress, nutrient availability has the potential to affect energy metabolism and protein synthesis as well as endocrine, nervous and immune systems [109]. The overall metabolic effect of the hormonal changes is increased metabolism, which mobilizes substrates to provide energy sources, and a mechanism to retain salt and water and maintain fluid volume, cardiovascular homeostasis and immune system response [109]. The extent to which a certain nutrient regulates the stress response depends on its duration, the athlete’s nutritional status as a whole, the type and intensity of the exercise, the physiological status, and the gut microbiota composition and function [110]. Other factors that make nutritional assessment difficult are the individual's genetic background and epigenetic profile [110]. Understandably, due to the considerable complexity of stress response in elite athletes (from leaky gut to catabolism and depression), defining standard diet plans is difficult. In general, many elite athletes are encouraged to consume high amounts of simple carbohydrates and protein and low amounts of fat and fiber in order to provide a quick source of energy while also avoiding potential digestive issues such as gas and distension that high fiber diets can sometimes cause [28]. Elite athletes’ dietary plans are also based on the consumption of certain micronutrients such as iron, calcium, amino acids, essential fatty acids and antioxidants [111], but rarely is the health of the gut microbiota ever considered.

Since diet strongly influences microbiota composition and function, modulation of the gut microbiota via nutritional treatments may improve the stress response in athletes and improve performance. It can be assumed that each dietary plan is probably accompanied by a simultaneous adjustment of the microbiota [112]. Short term consumption of a mostly animal or mostly plant diet can dramatically alter the microbiota community [26]. Another important consideration when designing personalized diets for athletes is to understand how the microbiome changes over time [8]. An initial bacterial community is established at birth, but develops as a person matures [8].

The nutritional strategies that may enhance exercise and/or training adaptations leading to improved health and performance are outlined in Table 2, along with the text below.

**Table 2:** Dietary Recommendations for elite athletes based on current evidence

Nutrient	Common recommended intake	Claimed benefits	Disadvantages	Recommendations
Carbohydrates	7 to 12 g/kg per day for athletes who train for more than 2 h/day [145]	Restore muscle and liver glycogen stores during intense exercise; Attenuate stress hormone levels and immunosuppression; Reduce fatigue and improve performance and mood [35].	Do not improve immune function nor do prevent decreased plasma glutamine concentrations after intense training [35]; Do not promote a healthy gut microbiota [21]	High carb intake from various sources, together with high protein ingestion may increase carbohydrate oxidation rates and attenuate energy depletion during competition [66]. Complex plant carbohydrates, and plant-based protein are recommended during training and resting periods to promote a healthy gut microbiota [16].
Protein	1.2 to 1.6 g/kg per day in the top elite athletes [41, 42]	Amino acids are spared for protein synthesis and are not oxidized in order to meet energy needs [42]. Adequate protein intakes enhance host immunity with particularly effects on the T cell system, resulting in decreased incidences of infections. Reduce fatigue and diet-dissatisfaction [35]	High-protein, low-carb diets before exercise reduces plasma glutamine concentrations post-exercise [44]. High animal protein intake can produce potentially toxic compounds in the gut [103]	Given the existing evidence, it is not recommended that elite athletes consume more than 1.2-1.6 g protein/kg.
Amino Acids				
Glutamine	There are no determined glutamine recommendations, though acute dosage of >20–30 g seem to be without ill effects in healthy adult humans [47]	An acute dose of oral glutamine 2 h before intense exercise may ameliorate stress-induced intestinal permeability, lower plasma endotoxins and be anti-inflammatory [65]	Acute glutamine supplementation taken during and after exercise in sufficient amounts to prevent the post-exercise fall in plasma glutamine concentrations have no effect on salivary IgA nor lymphocyte function [48]	Glutamine supplementation should depend on symptomatology (i.e. low plasma glutamine levels, leaky gut).
Branched chain amino acids (BCAA)	There are no established recommendations for BCAA supplementation, though they supposedly improve exercise performance while increasing muscle protein synthesis.	Leucine supplementation can greatly increase leucine and total BCAA concentrations and improve endurance performance [49] and muscle protein synthesis. BCAA may mediate effects of fatigue during exercise by modifying certain brain neurotransmitters [50]	While BCAA do compete with free tryptophan to cross the blood–brain barrier, evidence that increased brain 5-HT is driven by an increase in free tryptophan pools in blood is very weak. BCAA supplementation may be effective at reducing fatigue by increasing ammonia production	Due to the lack of evidence, no recommendation on the type or amount of BCAA athletes should take can be made.
Tyrosine, 4-hydroxyphenylalanine	No supplementation dose has been established. Many athletes may supplement with tyrosine as a way to balance tryptophan: tyrosine ratio as a way to reduce fatigue.	The acute consumption of tyrosine increases the ratio of tyrosine to other large neutral amino acids. Tyrosine supplements (150 mg/kg) might reduce adverse effects of acute stress [63].	Tyrosine ingestion does not influence time to exhaustion or several aspects of cognitive function while exercising in heat conditions [99].	Given the inconclusive results, it is not possible to define specific amino acid recommendations that may reduce central fatigue.
Fat and polyunsaturated fats	Fat consumption among athletes tends to be quite (15-30% of dietary energy) [56]. Increased fat metabolism during prolonged exercise may improve performance [111]. Fat intake of 30-50% of dietary energy may benefit endurance athletes [112] and improve energy [109]	Lipids attenuate intestinal inflammation, bacterial translocation and intestinal injury following intestinal hypoperfusion in athletes with digestive disturbances [113]. Post-exercise lipid ingestion may improve GI function and reduce the flu- like symptoms associated with endotoxemia by improving post-exercise splanchnic flow	High-fat diet microbiota can increase anxiety-like behaviour and neuro-inflammation and disrupt intestinal barrier function [114]. High-fat diets could be detrimental to immune function compared to high carbohydrate diets [85]. Omega-6 polyunsaturated fatty acids can negatively alter cell membrane fluidity and immune function during and after exercise [115]	The effects high fat diets have on exercise performance are equivocal, and there lacks information regarding stressed individuals. An optimal dosage of omega-3 polyunsaturated fatty acids seems to be approximately 1–2 g/d, at a ratio of EPA to DHA of 2:1 to reduce ROS and inflammation [117]
Vitamins and Antioxidants	Vitamins and other antioxidants are not normally increased in athletes, although some are recommended (vitamins C, E, β-carotene and polyphenols), to reduce free radical formation and lipid peroxidation [119]	Polyphenol supplementation with blueberry and green tea extracts increased the metabolites characteristic of gut bacteria polyphenol metabolism and ketogenesis in runners during recovery from 3-d heavy exertion [120]	Although no negative effects have been reported, athletes´ diets enriched with polyphenol extracts (blueberry and green tea), they do not mitigate the physiological stress of heavy exertion nor do they improve recovery speed [120].	Large doses of simple antioxidant mixtures or individual vitamins are not recommended and may be toxic. Athletes should obtain antioxidants from an increased consumption of fruits and vegetables [95].
Fiber	Adequate fiber intake is 14 g total fiber per 1,000 kcal, or 25 g for adult women and 38 g for adult men, based on research demonstrating protection against various diseases [121].	Low dietary fiber consumption is associated with lower microbiota diversity, fewer anti-pathogenic bacteria and less SCFA production [146], which may lead to inflammation [122] and less sympathetic nervous system stimulation [123]	Eating a high fiber diet before an intense training or competition could produce GI upset such as distension, gas and bloating [127]	Athletes should increase their intake of plant foods (e.g. whole grains, legumes, vegetables, fruits, and nuts) hours prior to or after training and consume less processed foods high in added sugar, refined carbohydrates and fat [121]
Probiotics	Probiotic supplementation is highly variable depending on the strain, microbial composition and metagenome. Due to the great diversity of the human microbiome, there have not been specific established dietary recommendations for probiotic supplementation for athletes.	Fermented foods enriched with *Lactobacillus* sp. and *Bifidobacteria* sp [129] can result in specific changes in gut microbiota activity, improving stress-induced symptoms such as depression, mood disturbance as well as digestive issues [130].	*Lactobacillus acidophilus*, *Lactobacillus casei* and *Bifidobacterium bifidum* had beneficial effects on depression in major depressive patients [132]. B*ifidobacterium longum* R0175 (PF) can reduce anxiety and free cortisol levels [133]. *Lactobacillus helveticus* also reduces anxiety [133] and plasma ACTH and corticosterone concentrations in response to stress in rats and can restore hippocampal serotonin (5-HT) and NE levels [74].	*Bifidobacterium* strains, which is common in the gut flora of many mammals, including humans, have generated the best results [134] though more research is needed to better understand the gut-brain axis.

#### Carbohydrates

There is no doubt that adequate carbohydrate consumption is essential for heavy training schedules and successful athletic performance [113]. Dietary carbohydrate intake ranges from 7 to 12 g/kg per day and fat intake is usually < 1 g/kg of body mass per day (<20% of total calories consumed) for athletes who train for more than 2 h/day [111]. Carbohydrates restore muscle and liver glycogen stores during prolonged periods of intense exercise [113], attenuate increased stress hormone levels, such as cortisol, and can limit the immunosuppression associated with high intensity exercise [113]. High carbohydrate diets (8.5 g/Kg/d; 65% total energy intake) [114] and eating carbohydrates *ad libitum* during intense periods of training can reduce fatigue and improve physical performance and mood [115]. However, a high carbohydrate diet does not improve immune function nor it does prevent decreased plasma glutamine concentrations after heavy periods of training [105, 113]. Moreover, the combination of glucose and fructose have shown to be beneficial since it resulted in higher carbohydrate oxidation rates than the ingestion of a single carbohydrate, attenuating the depletion of endogenous energy stores during exercise and stimulating repletion of these stores during acute post exercise recovery [64]. Nevertheless, diets high in simple and refined carbohydrates do not promote a healthy gut microbiota composition nor do they produce beneficial SCFA [116]. More studies are warranted to understand the capacity of the microbiota to extract nutrients from the diet and in including metabolic changes in the host, such as increased fatty acid oxidation in muscle and increased triglyceride storage in the liver during exercise.

#### Protein and essential amino acids

The daily protein requirement is approximately doubled in athletes compared to the sedentary population [113]. Protein intake necessary ranges from 1.2 to 1.6 g/kg per day in the top sport elite athletes [117, 118] so that amino acids are spared for protein synthesis and are not oxidized to assist in meeting energy needs [118]. Inadequate protein intake impairs host immunity with particularly detrimental effects on the T cell system, resulting in increased incidences of infections [113]. Prolonged exercise is also associated with a fall in the plasma concentration of glutamine and it has been hypothesized that such a decrease could impair immune function and increase susceptibility to infection and leaky gut in athletes [103]. Stress, fatigue and diet-dissatisfaction were higher during moderate-protein, moderate-fat diets (1.6 g protein/kg and 15.4% of calories in fat) compared to a high-protein and low-fat diet (2.8 g protein/kg and 36.5% of calories in fat) [119]. Consuming a high-protein, low-carbohydrate diet for several days prior to exercise results in a lower plasma glutamine concentration after exercise [120]. However glutamine supplements have received little support from well-controlled scientific studies in healthy, well-nourished athletes. There are no determined glutamine recommendations, though acute dosage of approximately 20–30 g seem to be without ill effects in healthy adult humans [121]. On the other hand, an acute dose of oral glutamine 2 h before intense exercise ameliorates stress-induced intestinal permeability and lowered plasma endotoxins and may produce anti-inflammatory effects and is a common supplement used to repair and restore intestinal barrier function [122]. Acute glutamine supplementation taken during and after exercise in sufficient amounts to prevent the post-exercise fall in the plasma glutamine concentration has no effect on salivary IgA nor lymphocyte function [120]. Therefore, we conclude that glutamine supplementation should depend on symptomatology (i.e. low plasma glutamine levels, leaky gut).

Currently, there are no established recommendations for BCAA supplementation, though they supposedly improve exercise performance while increasing muscle protein synthesis and reducing its soreness. Muscle protein synthesis has been shown to be 33% greater after consumption of leucine enriched essential amino acids than after consumption of essential amino acids [123]. Leucine supplementation resulted in significant increases in plasma leucine and total branched chain amino acids concentrations and improved endurance performance and upper body power, affecting the plasma tryptophan: BCAA ratio [124]. Supplementation of BCAA have also been used to mediate effects of fatigue during exercise by modifying brain neurotransmitters production such as 5-HTP, dopamine and noradrenaline [95]. While BCAA do compete with free tryptophan to cross the blood–brain barrier, evidence that increased brain 5-HTP is driven by an increase in free tryptophan pools in blood is very weak. Due to the lack of evidence, no recommendation on the type or amount of BCAA athletes should take can be made.

As explained above, the new central fatigue hypothesis states that fatigue sets in when serotonin levels are elevated and dopamine levels decrease, which could be why many athletes take tyrosine supplements to prevent its depletion, though no recommended supplementation dose has been established. Tyrosine, or 4-hydroxyphenylalanine, can be synthesized in the body from phenylalanine, and is found in many high- protein foods such as soy products, chicken, turkey, fish, peanuts, almonds, avocados, milk, cheese, yoghurt and sesame seeds [125]. A series of studies have indicated that tyrosine supplements (150 mg/kg) reduce many of the adverse effects of various types of acute stress [126]. Glutamine–arginine–citrulline supplementation has been recommended if perfusion of the gut is one of the main problems athletes face [127]. However, tyrosine ingestion does not influence time to exhaustion or several aspects of cognitive function while exercising in heat conditions [128]. Given the inconclusive results, it is not possible to define specific amino acid recommendations that may reduce the central fatigue syndrome.

While athletes may require a higher protein intake, high protein diets can affect the microbiota composition and function through amino acid fermentation in the colon that produces undesirable metabolites (e.g. phenol, hydrogen sulfide and amines) and urea and tend to lead to higher fecal pH (reviewed by Windey et al [129]). Intense exercise has been shown to increase plasma urea levels due to protein catabolism and the continual stress of training [130]. Most of host-produced urea is hydrolyzed in the lumen of the large intestine into NH_3_ and nitrogen through bacterial urease activity [131]. NH_3_ can be used by the bacteria for their own metabolism and protein synthesis [131]. Alternatively, it is absorbed by the colonocytes, transformed to urea in the liver and excreted in urine [129]. Therefore, the high levels of urea commonly seen in athletes could change the microbiota composition due to availability of nitrogen for their own proliferation and metabolism. Additionally, bacteria such as those from the Bacteroides phylum, ferment certain amino acids and proteins that result in BCAA along with potentially toxic byproducts such as ammonia, amines, volatile sulfur compounds [74], as well as phenolic compounds, indolic compounds [132], sulphides and organic acids [133]. P-cresol, phenols, certain amines and hydrogen sulfide have a known role in irritable bowel disease, colon cancer, increased intestinal permeability, inflammation, DNA damage and more [36].

It is important to note that while bacteria do ferment amino acids, they metabolize animal and vegetable proteins differently [134]. As mentioned above, each dietary plan is accompanied by a simultaneous adjustment of the microbiota composition and function [112]. Consequently, the microbiota composition of vegans, vegetarians, omnivores and diets high in red meat consumption differ greatly [134]. Koeth et al [134] reported the gut microbiota from mice that were supplemented with L-carnitine had an altered cecum microbiota composition that metabolized trimethylamine into trimethylamine-N-oxide (TMAO), which is associated with atherosclerosis. The authors concluded that diets high in red meat lead to a higher risk in cardiovascular disease due to microbiota-dependent production of TMAO. In line with this, Toden et al [135] fed rats with a diet containing 15% of casein, 25% of casein or 25% cooked lean red beef, each with or without the addition of 48% high amylose maize starch for four weeks. High dietary casein caused a 2-fold increase in colonic DNA damage compared to a low casein diet and reduced the thickness of the colonic mucus layer by 41%. High levels of cooked meat caused 26% greater DNA damage than the high casein diet but reduced mucus thickness to a similar degree to casein [135]. Despite this, adding resistant starch to these high protein diets nullified the negative effects of high protein consumption [135], further illustrating the importance of consuming adequate dietary fiber for gut and overall health. Another study examined the effects of protein type, and protein level on large intestine health in rats [136]. Lower levels of cecal BCAA were found in rats who ate a lower protein diet (14% of total energy) than those who consumed a high protein diet (20% of total energy) [136]. These authors also showed that plant-based protein proved to be beneficial compared to animal protein, where potato protein concentrate (PPC) consumption positively impacted colonic health by reducing enzymatic activity of β-glucuronidase, which is a biomarker for the risk of carcinogenesis [136]. The rats that ate PPC and low protein diets also presented deeper cecal crypts, illustrating that they had more cell proliferation and renewal which is necessary for epithelial repair [136]. While the amounts of Bacteroides and Firmicutes associated with vegan, vegetarian and omnivore diets from these studies and others [137, 138] are conflicting, it can be concluded that eating vegetables, fiber and/or resistant starch along with animal protein seems to diminish the negative effects of the potentially harmful byproducts from amino acids fermented by the gut microbiota.

#### Fats and polyunsaturated fatty acids

Fat consumption among athletes tends to be quite low, comprising between 15-30% of the dietary energy [139]. An increase in fat metabolism (30-50% dietary energy) during prolonged exercise may have a glycogen sparing effect and may improve endurance performance [140] and health [141]. In fact, high-lipid enteral nutrition has been shown to attenuate intestinal inflammation, bacterial translocation and intestinal injury following intestinal hypoperfusion with digestive disturbances [142]. On the other hand, high-fat diet can lead to increased anxiety-like behavior with selective disruptions in exploratory, cognitive, and stereotypical behavior, neuroinflammation disrupted markers of intestinal barrier function, as well as increased circulating endotoxin and lymphocyte expression compared to mice with control diet [143]. In humans, Pedersen et col [144] suggested that diets rich in fat (62% of dietary energy) could be detrimental to immune function compared to high carbohydrate diets (65% of dietary energy). These authors compared 10 untrained young men fed with carbohydrate-rich diet and 10 subjects fed with fat-rich diet during an endurance training of 3–4 times a week for 7 weeks [144]. Blood samples for immune monitoring were collected before and at the end of the study. NK cell activity had increased in the group that had the carbohydrate-rich diet [from 16% to 27%] and decreased in the group that had the fat-rich diet [from 26% to 20%] in response to training [144]. NK cells represent a critical component of the innate immune defense, recognizing transformed cells independently of antibodies or major histocompatibility complex restriction [145]. Thus the NK cell activity (the ability of NK cells to lyse a certain number of tumor target cells) was lower in athletes fed with high-fat diets [144]. Little is known about the mechanisms behind NK protection during exercise, but very recently, Pedersen et al [145] have demonstrated in tumor-bearing mice that NK cell infiltration was significantly increased in tumors from running mice, whereas depletion of NK cells enhanced tumor growth and blunted the beneficial effects of exercise.

Omega-6 polyunsaturated fatty acids can alter cell membrane fluidity and indirectly affect immune function including reduced IL2 production and suppressed mitogen-induced lymphocyte proliferation producing potentially an undesirable immune function during and after exercise [146]. However, an optimal dosage of omega-3 polyunsaturated fatty acids of approximately 1–2 g/d, at a ratio of eicosapentaenoic acid to docosahexaenoic acid of 2:1 may decrease the production of inflammatory eicosanoids, cytokines and ROS during exercise [147]. As of now, it is difficult to make any firm recommendations for athletes regarding the amount and duration of omega-3 supplementation due to conflicting results.

Currently, the effects that consuming a high fat diet have on subsequent exercise performance are equivocal, and there lacks information regarding stressed individuals [148]. On top of that, consumption of diets high in fat and calories is associated with chronic “low-grade” systemic inflammation, increased intestinal permeability and plasma LPS together with a decrease in total bacterial density and an increase in the relative proportion of Bacteroidales and Clostridiales orders [149]. Thus, consumption of a high-fat diet may also induce unfavorable changes in the gut microbiota [149].

#### Vitamins and antioxidants

Athletes are not normally supplemented with vitamins and other antioxidants, although it has been recommended that athletes should consider increasing their intakes of antioxidants, such as vitamins C, E, β-carotene and polyphenols, in order to reduce ROS formation and lipid peroxidation [150]. Polyphenol supplementation with blueberry and green tea extracts (as an ibuprofen substitute) did not alter the established inflammation and oxidative stress, but increased amounts of metabolites characteristic of gut bacteria polyphenol metabolism (e.g., hippurate, 4-hydroxyhippuric, 4-methylcatechol sulfate) and ketogenesis in runners during recovery from 3-d heavy exertion [151]. Although no negative effects have been reported, athletes’ diets enriched with polyphenol extracts (blueberry and green tea) have not mitigated the physiological stress of heavy exertion nor did it improve recovery speed [151]. Supplementation of individual micronutrients or consumption of large doses of simple antioxidant mixtures is not recommended [122]. Consuming mega doses of individual vitamins (not uncommon in athletes) is likely to do more harm than good, because most vitamins function mainly as coenzymes in the body [122]. Once these enzyme systems are saturated, the vitamin in free form can have toxic effects [122]. Therefore, athletes should obtain complex mixtures of antioxidant compounds from increased consumption of fruits and vegetables.

#### Fiber

The Academy of Nutrition and Dietetics has recently established that adequate fiber intake is 14 g total fiber per 1,000 kcal, or 25 g for adult women and 38 g for adult men, based on research demonstrating protection against coronary heart disease among diseases [152]. Low dietary fiber consumption is associated with lower microbiota diversity, less SCFA production [112] and fewer anti-pathogenic bacteria [153], all of which may have harmful long-term consequences for the host [112]. Acetate, propionate and N-butyrate are mediators of the colonic inflammatory response [154], stimulate sympathetic nervous system [155] and mucosal serotonin release [156]. Most athletes do not consume sufficient fiber and resistant starch [28] that feed commensal bacteria that produce beneficial byproducts for host metabolism and homeostasis such as SCFA and active neurotransmitters. For instance, endurance-trained athletes consumed less than 25 g · per day [157], whereas the fiber intakes of high level soccer players (age range: 15–17 years) were ~16 g per day [158]. Dietary habits of Flemish adolescent track and field athletes showed that fiber intake (girls 23.7 +/− 7.9 g; boys 29.1 +/− 11.2 g) was far below the Academy of Nutrition and Dietetics’s recommendations [159]. Athletes can achieve adequate dietary fiber intakes by increasing their intake of plant foods (e.g. whole grains, legumes, vegetables, fruits and nuts) while concurrently decreasing energy from processed foods high in added sugar, refined carbohydrates and fat during the recovery period and training period, as eating a high-fiber diet before an intense training or competition could produce GI upset such as distension, gas and bloating [160]. Additionally, dietary fiber and high consumption of plant-based foods appears to inhibit the bacteria from producing harmful metabolites from proteins, emphasizing the importance of eating adequate complex carbohydrates to maintain gut microbiome carbohydrate fermentation [36].

#### Probiotics

There is now a reasonable body of evidence that shows that consuming probiotics regularly may positively modify the gut microbiota’s population and structure and may influence immune function as well as intestinal epithelium cell proliferation, function and protection in individuals who follows exercise (reviewed by Mach et al [23]). The consumption of prebiotics (fermented dietary ingredients including fructans and oligosaccharides) and fermented foods enriched with *Lactobacillus* sp. and *Bifidobacteria* sp can result in specific changes in gut activity [161], suggesting that diet may provide a feasible means of microbiota modification. Additionally they might improve stress-induced symptoms such as depression, mood disturbance and other digestive issues such as inflammation [162, 163]. Probiotic supplementation is highly variable depending on the strain and microbiota composition and thus there have not been specific established dietary recommendations for dosages nor strains in athletes [23]. Yogurt supplemented with beneficial bacterial strains are already being used to help treat some GI disorders such as inflammation and epithelial barrier function restoring [23]. For instance, the strain *Lactobacillus rhamnosus* CNCMI–4317 was able to regulate multiple pathways including cellular function and maintenance, lymphoid tissue structure and development, immune system response as well as lipid metabolism in epithelial cells [163]. However, there is little data about how probiotics affect the human behavior and gut-grain axis. More research is needed to better understand how probiotics can ameliorate depressive symptoms. However, patients with major depressive order who supplemented with *Lactobacillus acidophilus*, *Lactobacillus casei* and *Bifidobacterium bifidum* for 8 weeks had beneficial effects on depression, insulin and glutathione concentration [164]. *Bifidobacterium longum* R0175 taken for 30 days reduced anxiety-like behaviors and stress levels (indicated by urinary free cortisol levels), and significant improvements in anxiety and depression were observed [165]. Currently, *Bifidobacterium* strains, which are common in the gut microbiota of many mammals, including humans, have generated the best results [166]. In Wistar rats, 14-day administration of combined *Lactobacillus helveticus* and *Bifidobacterium longum* reduced anxiety-like behavior in the defensive marble burying test possibly because the probiotic reduced HPA acids and ANS activity [165]. *Lactobacillus farciminis* and *Lactobacillus helveticus* NS8 have been shown to decrease plasma ACTH and corticosterone concentrations in response to stress in rats [167, 168], as well as restore hippocampal serotonin and NE levels and decrease neuroinflammation [169]. Yet, we still need a lot more research into the mechanisms by which gut bacteria interact with the brain and may be able to modify the mood, fatigue, depression and overall health in our athletes.

It is possible that inoculating elite athletes’ microbiota with different species may be necessary to restore important functions of the gut and brain. Because the gut microbiota regulates numerous facets of human biology it is important to establish specific diets that could be used in adjunct or sole therapy for microbiota enrichment in athletes.

It is clear then that the interaction between athlete’s diet and exercise needs to be further studied in order to better assess the contributions of diet and microbial activities in athletic performance and stress-related symptoms. Modifying athletes’ diets in a way in which they positively impact the activities of their gut microbiota through newly recognized inter-kingdom axes of communication such as the gut-brain axis may also benefit sport performance.

## Conclusions

Exercise-induced stress modifies stress and catabolic hormones, cytokines and gut microbial molecules, which might result in gastrointestinal disturbances, anxiety, depression, and underperformance. The gut microbiota has fundamental roles in many aspects of human biology, including metabolism, endocrine, neuronal and immune function. In murine models, intense exercise-induced stress exacerbated intestinal inflammation and clinical outcomes through a decrease of *Turicibacter* spp. and increase of *Ruminococcus gnavus, Butyrivibrio* spp., *Oscillospira* spp., and *Coprococcus* spp*.* In light of these preliminary results, changes in athletes mood and gastrointestinal function could reflect the underlying interaction between the gut microbiota and gut-brain axis during times of intense physical stress.

Appropriate nutritional choices (i.e. avoiding fat and fiber) have been recommended to reduce the risk of GI discomfort in elite athletes by ensuring rapid gastric emptying, water and nutrient absorption and adequate perfusion of the splanchnic vasculature before competitions. However, the lack of complex carbohydrates in elite athletes’ diets may negatively affect the gut microbiota composition and function in the long run. The gut and the microbiota are important organs for athletic performance because they are responsible for the delivery of water, nutrients and hormones during exercise. Therefore, an increased consumption of complex plant polysaccharides should be promoted to help maintain gut microbiota diversity and function. It should also be noted that high animal protein consumption during resting days and training should be reduced because it may negatively affect the gut microbiota (e.g. production of potentially toxic byproducts such as amines and volatile sulfur compounds). Supplementing the diet with prebiotics and/or probiotics that stimulate the expansion of specific microorganisms such as *Bifidobacteria* and *Lactobacillus* and beneficial metabolites such as SCFA to improve the metabolic, immune and barrier function can be a therapy for athletes. With this in mind, the modulation of the microbiota and its fermentation capacity may provide the scientific basis for designing diets aimed at improving performance by enhancing healthy microbiota’s metabolites during exercise and limiting those that produce toxic metabolites that may made worsen the consequences of stress.

## Abbreviations

5-HTP: 5-hydroxytryptamine
AADC: Amino acid decarboxylase
ACTH: Adrenocorticotropin
ANS: Autonomic nervous system
AVP: Arginine vasopressin
BCAA: Branched-chain amino acids
BNST: Bed nucleus of the stria terminalis
CRH: Corticotropin-releasing hormone
EC: Enterochromaffin cells
EN: Epinephrine
ENS: Enteric nervous system
GABA: Gamma aminobutyric acid
GCs: Glucocorticoids
GF: Germ-free
GI: Gastrointestinal
GR: Glucocorticoid receptor
HPA: Hypothalamus-pituitary-adrenal axis
IDO: Indoleamine 2,3- dioxygenase 1
IFNα: Interferon alpha
IFNγ: Interferon gamma
IL1β: Interleukin 1 type β
IL2: Interleukin 2
IL6: Interleukin 6
LPS: Lipopolysaccharide
MR: Mineralocorticoid receptor
NE: Norepinephrine
NFKβ: Nuclear factor kappa-light-chain-enhancer of activated B cells
NPY: Neuropeptide Y
OTS: Overtraining syndrome
PPC: Potato protein concentrate
PVN: Paraventricular nucleus of the hypothalamus
ROS: Reactive oxygen species
SAM: Sympathetic-adrenomedullary system
SCFA: Short chain fatty acids
SPF: Specific pathogen free
TDO: Tryptophan-2,3-dioxygenase
TLR4: Toll-like receptor 4
TMAO: Trimethylamine-N-oxide
TNFα: Tumor necrosis factor alpha
TPH: Tryptophan hydroxylase
V_O2max_: Maximal oxygen uptake
